# Colonic lymphomatous polyposis mantle cell lymphoma: a case report and review of literature

**DOI:** 10.1186/s13256-024-04533-z

**Published:** 2024-05-03

**Authors:** Toukilnan Djiwa, B. B. S. Koui, N. A. Aman, Z. I. Coulibaly, M. Kouyate, K. E. Kouame

**Affiliations:** 1 Department of Pathological Anatomy, Teaching Hospital of Treichville, Abidjan, Ivory Coast; 2 Department of Pathological Anatomy, Teaching Hospital of Bouaké, Bouake, Ivory Coast; 3 Department of Pathological Anatomy, Teaching Hospital of Lomé, BP 1515, Lomé, Togo

**Keywords:** Polyposis, Lymphoma, Mantle cell lymphoma, Colon

## Abstract

**Introduction:**

Mantle cell lymphoma is a rare lymphoma of the gastrointestinal tract that may present as multiple lymphomatous polyposis. We report a case of lymphomatous polyposis with a review of the literature.

**Case report:**

A 56-year-old man of Black ethnicity and Ivorian nationality with no relevant past medical history, consulted for a sudden onset symptoms of gastrointestinal obstruction, which evolved over 2 days. Macroscopic examination revealed the presence of multiple polyploid formations of the colonic mucosa. Histology showed diffuse lymphomatous proliferation of submucosa consisting off small lymphoid cells with a hyperchromatic crenelated nucleus, suggesting lymphomatous polyposis. Immunohistochemical examination showed expression by the tumor cells of antibodies to CD20, CD5, Bcl2, and cyclin D1. They did not express antibodies to CD10 and CD23. The Ki67 proliferation index was 25%. We have thus retained the diagnosis of mantle cell lymphomatous polyposis.

**Conclusion:**

Multiple lymphomatous polyposis is a rare entity characterized by the presence of numerous gastrointestinal polyploid lesions sometimes involving several segments of the gastrointestinal tract. Typical lymphoma presenting as lymphomatous polyposis is mantle cell lymphoma; although, other tumors may have this aspect.

## Introduction

Non-Hodgkin lymphoma (NHL) of the digestive tract is the most common extraganglionic lymphoma. Approximately 15–30% of primary extraganglionic lymphomas occur in the gastrointestinal tract, accounting for 1–10% of all gastrointestinal cancers [1–3]. Mucosa-associated lymphoid tissue (MALT) lymphoma is the most common histological subtype in the stomach, mantle cell lymphoma (MCL) in the terminal ileum, jejunum, and colon, T cell lymphoma is associated with enteropathy in the jejunum, and follicular lymphoma in the duodenum with geographic variation in distribution [4].

Mantle cell lymphoma is an aggressive B cell neoplasm characterized primarily by a monotonous proliferation of small-to-medium-sized lymphocytes coexpressing CD5, CD20, and cyclin D1 epitopes and frequently presents chromosomal translocations, t(11 14) (q13; q32) [5, 6]. Mantle cell lymphoma occurs more frequently in older males, representing approximately 4% of all lymphomas in Western countries [7]. Mantle cell lymphoma usually affects the gastrointestinal tract and may present as multiple areas of lymphomatous polyposis [8]. It is a rare type of gastrointestinal lymphoma, which infiltrates extensively into the intestine [9] and is characterized by the presence of several lymphomatous polyps along one or more segments of the gastrointestinal tract [2, 10]. Since the first description in 1961, only a few cases have been reported [11].

We report an unusual case of a patient with multiple areas of lymphomatous polyposis with extensive colorectal involvement and acute colonic obstruction, an atypical complication of this rare disease. On the basis of this case study, the pitfalls in the diagnosis and prognosis of gastrointestinal tract disorders of these polyposis presentations as well as the treatment options are discussed.

## Case report/observation

**Fig. 1:**
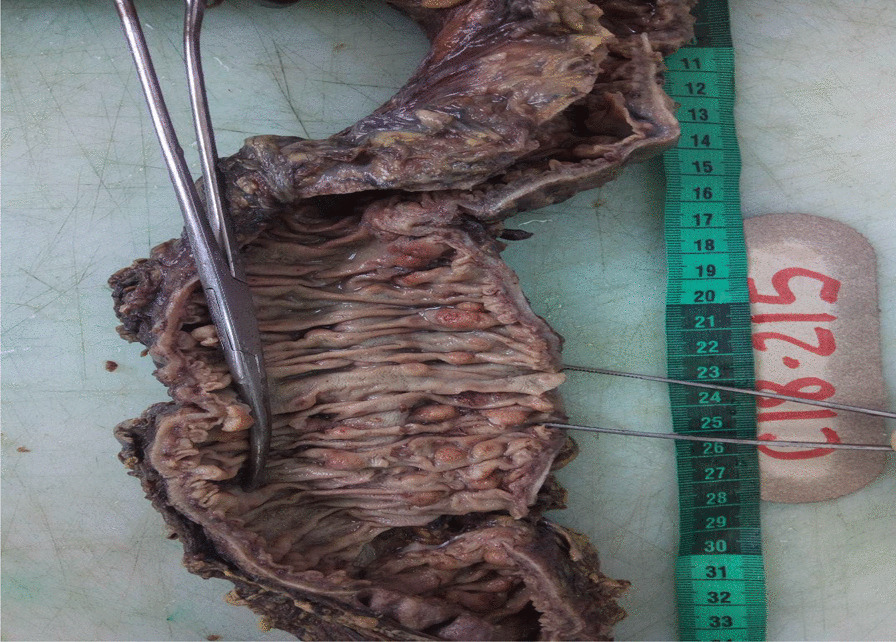
Multiple polypoid formations in the colonic lumen

**Fig. 2:**
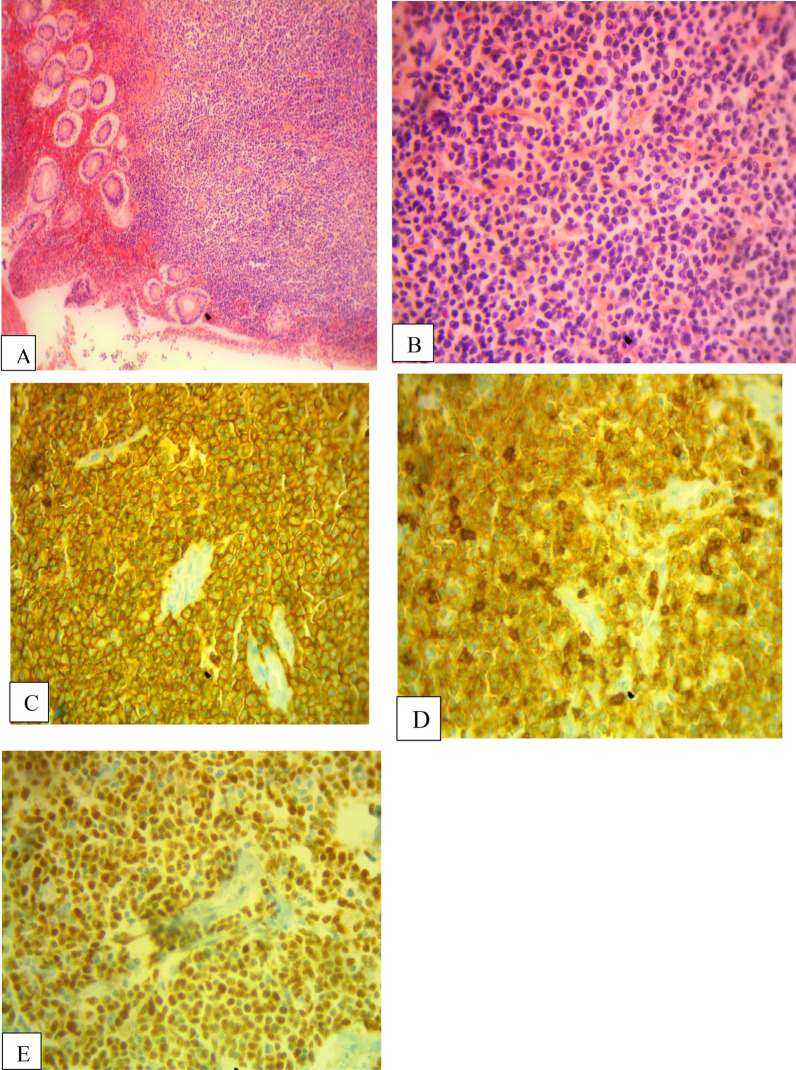
**A** Colon submucosa with infiltration by neoplastic lymphocytes (hematoxylin and eosin ×40). **B** Lymphoid cells with hyperchromatic nuclei (hematoxylin and eosin ×100). **C** The neoplastic lymphocytes are immunoreactive for CD20 (immunohistochemistry ×400). **D** The neoplastic lymphocytes are immunoreactive for CD5 (immunohistochemistry ×400). **E** The neoplastic lymphocytes are immunoreactive for cyclin D, which supports the diagnosis of mantle cell lymphoma (immunohistochemistry ×100)

This was a 56-year-old man of Black ethnicity and Ivorian nationality without any relevant past medical history, who consulted for abdominal pain with an abrupt obstipation of solids and gas for 2 days. The physical examination displayed a patient in good general condition. There was abdominal distension associated with tympanism. The rectal examination was normal, and examination of the other organs was normal. The lymph node areas were normal. Abdominal X-rays revealed dilated bowel loops with fluid levels consistent with descending colonic obstruction. An exploratory laparotomy was indicated. Numerous polyploid formations of the left colon and sigmoid were palpated, and the largest obstructed the colon lumen. A subtotal colectomy was performed with reconstruction via an ileocolostomy. There were no apparent signs of polyposis in the distal sigmoid and rectum. The surgical specimen was sent to the anatomic and cytologic pathology department of the teaching hospital of Treichville (Ivory Coast). Macroscopy of the surgical specimen showed multiple polyploid formations in the colonic mucosa (Fig. 1). Microscopic examination after standard paraffin embedding techniques identified a diffuse lymphoid proliferation of the submucosa, made of small lymphoid cells with a hyperchromatic crenelated nucleus. (Fig. 2A, B). These features suggested a lymphomatous polyposis of MALT lymphoma type, either lymphocytic lymphoma type or mantle cell lymphoma type. Immunohistochemical analysis showed the positivity of the tumor to CD20, CD5, Bcl2, and cyclin D1, while it was negative to CD10 and CD23. The Ki67 proliferation index was 25%. (Fig. 2C–E). In view of the morphological and immunohistochemical results, we made the diagnosis of primary mantle cell lymphomatous polyposis. The clinic-radiological assessment classified our patient at stage II according to the Ann Arbor classification modified by Cheson *et al*. (Table 1) [12]. The patient received chemotherapy according to the cyclophosphamide, doxorubicin, vincristine, and prednisone (CHOP) protocol associated with rituximab. After 1 year of follow-up, the patient has maintained a good general condition without recurrence.

**Table 1:** Ann Arbor classification modified by Musshoff

Stage I_E_	Involvement of one or more sites in the digestive tract without lymph node involvement
Stage II_E_	Involvement of one or more sites in the digestive tract and regional lymph nodes without extra-abdominal involvement Musshoff modification: Stage II1E, involvement of contiguous lymph nodes only Stage II2E, involvement of noncontiguous regional lymph nodes
Stage III_E_	Localized involvement of the digestive tract associated with lymph node involvement on either side of the diaphragm
Stage IV	Involvement of one or more extraganglionic and/or extra-abdominal organs ± associated lymph node involvement, particularly superficial

## Discussion

### Epidemiological features

Multiple lymphomatous polyposis is a rare and heterogeneous group of small B cell lymphomas. B cell polyposis neoplasms include mantle cell lymphoma, follicular lymphoma, and MALT lymphoma [3]. Initially described by Cornes *et al*. in 1961, multiple lymphomatous polyposis is characterized by the presence of diffuse proliferation of atypical lymphocytes presenting as multiple polypoid lesions in different segments of the gastrointestinal tract [13]. B cell lymphomas of the gastrointestinal tract are more common than T cell lymphomas because these polyps histologically originate from the mantle area of the lymphoid follicle of mucosal associated lymphoid tissue (MALT) [14]. Therefore, the typical lymphoma presenting as lymphomatous polyposis is mantle cell lymphoma, which has been reported with a frequency of up to 9% of all B cell lymphomas of the gastrointestinal tract [14]. The colon and the rectum are generally the most affected segments (90% of mantle cell lymphoma cases), followed by the small intestine, stomach, and duodenum [2, 15]. Regional lymph node involvement is common. Other possible extradigestive sites are the bone marrow, the peripheral lymph nodes, Waldeyer’s ring, the spleen, and the liver [14]. The patient was 56 years old. Lymphomatous polyposis frequently occurs in elderly patients, with a male predominance [16–18]. The etiopathogenesis is unknown, but a genetic background, exposure to previous chemotherapy, and exposure to ionizing radiation could contribute to the development of gastrointestinal mantle cell lymphomatous polyposis [13, 19].

### Clinical aspects

The clinical profile of mantle cell lymphomatous polyposis is heterogeneous. On the basis of the largest series of studies done by Ruskone-Fourmestraux *et al*., the main symptoms are abdominal pain, diarrhea, and hematochezia [2, 10, 20]; intestinal obstruction, exudative enteropathy, intestinal malabsorption, chylous ascites, and surgical abdomen are less frequent [2, 10]. Our patient was admitted for bowel obstruction.

### Histopathological aspects

Lymphomatous polyposis is more often mantle cell lymphoma. However, lymphomatous polyposis can originate from a follicular lymphoma or a MALT lymphoma [9]. Grossly, it is characterized by the presence of numerous polypoid lesions sometimes involving several segments of the gastrointestinal tract [14]. We found multiple polyploid formations in the colonic mucosa. Histologically, it is a monotonous proliferation of small-to-medium-sized lymphocytes with a hyperchromatic crenelated nucleus [5]. Other entities should be considered for differential diagnosis, including chronic lymphocytic leukemia, peripheral T cell lymphoma, and subtype of lymphocytic lymphoma (diffuse large B cell lymphoma and immune proliferative small intestinal disease) [10, 21–23]. Immunohistochemical examination confirmed the diagnosis of mantle cell lymphomatous polyposis. Mantle cell lymphoma expresses B cell markers (CD20 and CD79a), T cell markers (CD5), and cyclin D1. These markers are used in routine immunohistochemical testing to diagnose mantle cell lymphoma. However, CD10 is a marker for diffuse large B cell lymphoma, and CD23 is a marker for follicular lymphoma. Both are generally negative in mantle cell lymphoma [5, 6]. In our patient, immunohistochemical analysis revealed positive immunostaining for anti-CD20, anti-CD5, anti-Bcl2, and anti-cyclin D1 antibodies. In contrast, there was no immunostaining for anti-CD10 and anti-CD23 antibodies. The Ki67 was estimated to be 25%. Thus, positivity of CD20, CD5, Bcl2, and cyclin D1 led to the diagnosis of mantle cell lymphomatous polyposis.

### Therapeutic and prognostic aspects

Patients under 65 years of age with good general condition initially benefit from an intensive immunochemotherapy regimen (R-CHOP) combining rituximab with cyclophosphamide, doxorubicin, vincristine, and prednisone [24–26]. Rituximab monotherapy, a chimeric monoclonal antibody that specifically binds to the CD20 antigen, has response rates of 30%. When it is combined with anthracycline, the response rate can increase to more than 90% [26–28]. Standard immunochemotherapy with R-CHOP reduces lesions, and the initial response to treatment is 94% [24–26]. Unlike most B cell lymphomas, remissions of mantle cell lymphomatous polyposis are short [26, 27].

Mantle cell lymphoma has one of the poorest prognoses of all types of non-Hodgkin lymphomas, with a median survival time less than 3 years [2, 10, 22] and frequent relapses [3, 29]. Recent studies have suggested a role for bendamustine and rituximab, particularly if the patient’s functional status precludes a more aggressive chemotherapy regimen [30, 31]. The main treatment for mantle cell lymphoma is chemotherapy. Other treatments may include targeted therapy, biological therapy, radiotherapy, and stem cell transplantation [31]. Our patient received cyclophosphamide, doxorubicin, vincristine, and prednisone (CHOP) chemotherapy in combination with rituximab. After 1 year of follow-up, the patient has maintained a good general condition, without recurrence.

## Conclusion

Multiple lymphomatous polyposis is a rare and heterogeneous group of small B cell lymphomas, including mantle cell lymphoma, follicular lymphoma, and MALT lymphoma. It is defined by the presence of diffuse proliferation of atypical lymphocytes presenting as multiple polyploid lesions in different segments of the gastrointestinal tract. The typical lymphoma with lymphomatous polyposis is mantle cell lymphoma, described by overexpression of the anti-cyclin D1 antibody. It mostly occurs in elderly male patients.

## Data Availability

All data supporting the conclusions of this article are included in the manuscript.

## Abbreviations

NHL: Non-Hodgkin lymphoma
MCL: Mantle cell lymphoma
MALT: Mucosa-associated lymphoid tissue
R-CHOP: Rituximab, cyclophosphamide, doxorubicin, vincristine, and prednisone
